# Protein Malnutrition Impairs the Immune Response and Influences the Severity of Infection in a Hamster Model of Chronic Visceral Leishmaniasis

**DOI:** 10.1371/journal.pone.0089412

**Published:** 2014-02-25

**Authors:** Eugenia Carrillo, Mª Angeles Jimenez, Carmen Sanchez, Joana Cunha, Camila Marinelli Martins, Anaiá da Paixão Sevá, Javier Moreno

**Affiliations:** 1 WHO Collaborating Centre for Leishmaniasis, Centro Nacional de Microbiología, Instituto de Salud Carlos III, Madrid, Spain; 2 Departamento Medicina y Cirugía Animal, Facultad de Veterinaria, Universidad Complutense de Madrid, Madrid, Spain; 3 Instituto de Biología Molecular e Celular, Instituto de Ciências Biomédicas Abel Salazar e Faculdade de Medicina, Universidade do Porto, Porto, Portugal; 4 Departamento Medicina Veterinária Preventiva e Saúde Animal, Faculdade de Medicina Veterinária e Zootecnia da Universidade de São Paulo, São Paulo, Brazil; INRS – Institut Armand Frappier, Canada

## Abstract

Leishmaniasis remains one of the world's most devastating neglected tropical diseases. It mainly affects developing countries, where it often co-exists with chronic malnutrition, one of the main risk factors for developing the disease. Few studies have been published, however, on the relationship between leishmaniasis progression and malnutrition. The present paper reports the influence of protein malnutrition on the immune response and visceral disease development in adult hamsters infected with *Leishmania infantum* fed either standard or low protein diets. The low protein diet induced severe malnutrition in these animals, and upon infection with *L. infantum* 33% had severe visceral leishmaniasis compared to only 8% of animals fed the standard diet. The infected, malnourished animals showed notable leukocyte depletion, mild specific antibody responses, impairment of lymphoproliferation, presence of parasites in blood (16.67% of the hamsters) and significant increase of the splenic parasite burden. Animals fed standard diet suffered agranulocytosis and monocytopenia, but showed stronger specific immune responses and had lower parasite loads than their malnourished counterparts. The present results show that protein malnutrition promotes visceral leishmaniasis and provide clues regarding the mechanisms underlying the impairment of the immune system.

## Introduction

Malnutrition is a serious health problem that remains common in many parts of the world. Protein energy malnutrition is the most frequent form of malnutrition, and globally affects some 800 million people, including over 150 million children under 5 years of age, most of them in developing countries [1]. Much of the excess morbidity and mortality associated with malnutrition is owed to the impairment of sufferers' defence mechanisms, which predisposes them to infectious diseases [2]. Leishmaniasis, a vector-transmitted, poverty-related disease – the second and fourth most important among tropical diseases in terms of mortality and morbidity rates [3] – is commonly encountered where protein malnutrition is also prevalent. Visceral leishmaniasis (VL), the most severe clinical manifestation, is generally fatal if not treated.

Although there is strong evidence that malnutrition is one of the major factors influencing the outcome of visceral leishmaniasis [4], [5], few studies have measured the effects of this condition on the development of the disease. Anstead et al. (2001) reported that mice fed a protein-, iron- and zinc-deficient diet, were at increased risk of visceral disease following intradermal inoculation into both hind footpads with *L. donovani* [6]. Malafaia et al. (2009) also described that protein-deficient diets increased splenic parasitism in a BALB/c model of infection after intravenous challenge with *L. chagasi* [7]. Although it is clear that protection against *L. infantum* is dependent upon cellular immunity [8], which is known to be diminished by protein calorie malnutrition [9], the specific, cell-mediated mechanisms compromised in malnourished persons with visceral leishmaniasis remain unclear. In fact, the impact of infection or protein malnutrition on the host immune response, as well as the progression of the infection towards chronic visceral disease, have never been addressed with standardized experimental models.

Unlike in certain murine models, in which *Leishmania* infection tends to be self-limiting [10], golden hamsters (*Mesocricetus auratus*) develop a progressive, severe disease similar to visceral leishmaniasis seen in humans and dogs [8], [11]. The effects of low protein and standard diets on certain parasitic infections have been reported in hamsters [12], but their effects on the course of chronic visceral leishmaniasis are unknown. The aim of the present work was to examine the relationship between protein malnutrition and visceral leishmaniasis in a hamster model. The results showed prolonged protein deprivation to be associated with a weakened immune response and severe visceral disease following *L. infantum* infection.

## Materials and Methods

### Ethics statement

The research ethics and animal welfare committee of the Instituto de Salud Carlos III (“Comité de Ética de la Investigación y de Bienestar Animal, Instituto de Salud Carlos III”) approved this study (Report CBA PA 77_2010) that was carried out in accordance with Spanish law on the protection of animals used for experimentation and other scientific purposes (Royal Decree 1201/2005 and Law 32/2007). The Spanish legislation is a transposition of the Directive 86/609/EEC.

### Parasites

*L. infantum* promastigotes (MCAN/ES/98/LLM-722, JPC strain) were grown in NNN medium and complete RPMI medium (RPMI1640, Gibco, Paisley, UK) supplemented with 100 UI/ml of penicillin, 100 µg/ml of streptomycin, 2 mM L-glutamine, 5×10^−5^ M 2-mercaptoethanol and 10% heat inactivated foetal calf serum (Lonza, Spain) as liquid phase for 2 weeks.

### Animals and infection

Forty-eight 12 week-old male golden hamsters (*Mesocricetus auratus*), purchased from Janvier (France), were randomly assigned to isocaloric diets (Harlan Laboratories, Indianapolis, Indiana) containing either 14% (TD.00168) or 3% (TD.97138) protein (Table 1) but identical micronutrient content. Quantity of food given to hamsters from 14% group was adjusted to the 3% group to confirm comparable feed consumption [13]. Once total body weights in the low protein diet group showed statistical differences with those fed standard protein diet (day 53), 12 hamsters from both diet groups were inoculated with 2×10^7^ promastigotes by intracardiac route (i.e., the 14%INF and 3%INF groups). The remaining, non-infected hamsters were maintained on the initially assigned diets (14%NI and 3%NI groups). At 113 days post-infection, all animals were anesthetized with isoflurane and euthanized by cervical dislocation.

**Table 1:** Nutrients and kilocalories in the 3% and 14% protein diets used in the study.

Nutrient	% by weight		% kilocalories	
	3% Diet	14% Diet	3% Diet	14% Diet
Protein	3.1	14.2	3.3	15.2
Carbohydrates	78.6	66.6	83.6	71.5
Fat	5.5	5.5	13.2	13.3
Kcalories/g	3.8	3.7		

Control diet (14% protein diet) and low protein diet (3% protein diet) were purchased from Harlan Laboratories. Both diets contained similar amounts of corn oil (54.3 g/kg in 3% diet, 53 g/kg in 14% diet), cellulose (62.43 g/kg in 3% diet, 56.23 g/kg in 14% diet), Teklad vitamin mix 40060 (10 g/kg), ethoxyquin antioxidant (0.01 g/kg), calcium (0.7%) and phosphorous (0.54%).

### Haematology and biochemistry

Blood samples were collected from all animals by cardiac puncture (under anaesthesia with isoflurane) on day 113 post-infection. 300 µL were placed in Ca^2+^-EDTA tubes for haematological, flow cytometry and PCR determinations. Haematocrit and total erythrocyte, leukocyte, lymphocyte and platelet (PLT) counts were recorded using an automated blood cell counter (VetABC, Scil, France). The mean corpuscular volume (MCV), mean corpuscular haemoglobin (MCH) and red blood cell distribution width (RDW) were then calculated. The remaining fraction of the blood samples was placed in sodium heparin tubes. The plasma was then isolated, frozen and stored for subsequent biochemical and immunological analysis. Plasma alanine aminotransferase (ALT), aspartate aminotransferase (AST), blood urea nitrogen (BUN), creatinine, alkaline phosphatase (ALkP), glucose, globulins and total protein concentrations were determined using a Biochemistry Serum Analyzer (IDDEX, Netherlands).

### Cell isolation and proliferation assay

PBMCs from blood collected in sodium heparin tubes were separated on a Ficoll-Hypaque density gradient (Lymphocyte Isolation Solution, Rafer, Spain) and washed twice in phosphate-buffered saline (PBS, pH7.4). These cells were then cultured in flat-bottomed 96-well plates (1×10^5^ cells/well) at 37°C for 5 days in RPMI medium. After further incubation with 10 µg/ml soluble *Leishmania* antigen (SLA) or 10 µg/ml concanavalin A (CONA) (Sigma) (performed in triplicate), the plates were pulsed with 5-bromo-2′ deoxyuridine (BrdU) and lymphocytes proliferation examined using the BrdU Cell Proliferation Assay Kit (GE Healthcare Life Sciences, UK) according to the manufacturer's instructions.

### Delayed type hypersensitivity response

The delayed type hypersensitivity (DTH) response was determined by inoculating the left forepaw pad of each hamster with 5×10^4^ formalin-inactivated promastigotes (Leishmanin, Institute Pasteur, Teheran). The right forepaw was injected with PBS and used as a control. Oedema/inflammation was assessed by measuring paw pad thickness in the dorsal-plantar axis at 48 h post-inoculation.

### Flow cytometry analysis

50 µl of blood (from the samples collected in the Ca^2+^-EDTA tubes) were incubated with rat anti-mouse CD4 (clone 520 GK1.5, eBioscience, UK) and mouse-anti-mouse/rat MHCII (I-Ek) (clone 14-4-4S, eBioscience, UK); these have previously been shown to identify hamster CD4+ T cells [14] and B cells respectively [15]. The cells were then fixed in 1% p-formaldehyde-PBS solution and analysed by flow cytometry using a FACSCalibur (Becton Dickinson, Spain).

### Enzyme immunoassay (ELISA) and immunofluorescence antibody (IFAT) tests

Maxisorp microtitre plates (Nunc, Roskilde, Denmark) were coated overnight with 1 µg SLA in carbonate buffer (1 mM Na_2_CO_3_, 28 mM NaHCO_3_, pH9.6) and blocked for 1 h at 37°C with 200 µL of 1% BSA and 0.1% Tween20 in PBS. Plates were washed three times using PBS containing 0.01% Tween20, and then incubated for 30 min with 100 µL of plasma diluted 1∶100 with dilution buffer (0.1% BSA and 0.1% Tween20 in PBS). The plates were then washed and incubated for 30 min with 1∶250 horseradish peroxidase-conjugated goat anti-hamster IgG (Abd Serotec, Oxford, UK). ELISA was revealed using o-phenylenediamine dihydrochloride tablets (Sigma, Spain), quenching with 50 µL of 1M H_2_SO_4_. IFAT was performed using *L. infantum* promastigotes (MCAN/ES/98/LLM-722). Plasma was assayed in two-fold serial dilutions from 1/20 to ≥1/640 in PBS to determine total IgG levels using FITC-conjugated goat anti-hamster IgG (Abd Serotec, Oxford, UK) diluted 1/100. Antigen-antibody reactions in dilutions >1/40 were considered positive.

### Histopathology

After collecting circulating blood by cardiac puncture, necropsies were performed on all hamsters at day 113 post-infection at the *Centro Nacional de Microbiologia*, *Instituto de Salud Carlos III* (n = 48). Sections from the liver, spleen, right and left kidney, skin, ear, forelimbs, tongue and diaphragm were collected from each animal, placed in 10% buffered formalin and processed for histopathological analysis at the histology laboratory of the *Hospital Clinico Veterinario Complutense* (Madrid, Spain). Fixed tissues were embedded in paraffin, sectioned (4 μm), and stained with haematoxylin-eosin (H-E) following standard laboratory procedures. All samples were examined under the light microscope. Inflammatory and degenerative lesions in each tissue were qualitatively described and scored semi-quantitatively according to their severity as either non-existent (0), mild (+), moderate (++) or severe (+++).

### DNA extraction and real time PCR (qPCR)

Blood, ear skin, mesenteric lymph nodes, bone marrow, liver and spleen were aseptically collected during necropsy. Bone marrow cells were isolated by flushing the bone marrow from the left femur with RPMI medium. Liver and spleen samples from each animal were individually homogenised through a 40 μm stainless steel tissue grinder in RPMI, and 1×10^6^ cells used for total DNA isolation. 200 µL of blood from the samples collected in the Ca^2+^-EDTA tubes were also used.

400 µL of NET10 buffer (10 mM NaCl, 10mM EDTA, 10 mM Tris-HCl pH 8.0) and 40 µL of 10% SDS were added to each sample following incubation at 70°C for 1 h and DNA purification using traditional phenol/chloroform extraction and ethanol precipitation. Total DNA was resuspended in 100 µL of distilled water and quantified using an ND-1000 UV-V Spectrophotometer (NanoDrop Technology, USA). *Leishmania* DNA was quantified using a LightCycler high speed thermocycler and the LightCycler FastStart DNA Master SYBR Green I kit (Roche Diagnostics, Spain) as previously described [16].

### Evaluation of the severity of *L.infantum* infection

The evaluation of haematological and biochemical values, humoral and cellular responses, parasite load and histopathological damage of spleen and liver were assessed for each hamster and a disease score was performed. Altered parameters compatible with visceral leishmaniasis were scored with the maximum grade. Visceral leishmaniasis status was recorded as: Mild, Moderate, and Severe.

### Statistical analysis

To analyse weight variations between the groups, the area under the curve for each animal was calculated and the results subjected to two-way ANOVA [17].

Haematological, biochemical, immunological, parasitological data and the disease score were tested for normality using the Shapiro-Wilk test. Since some parameters were not normally distributed, non-parametric tests were chosen to compare groups. When comparing 3%INF, 14%INF, 3%NI and 14%NI, Kruskal-Wallis one-way analysis of variance was used followed by pair wise comparison of groups using Mann-Whitney U test. When comparing two groups, Mann-Whitney U test was used. For the analysis of the score, two multiple comparisons were performed. First, we studied 3%INF, 14%INF, 3%NI and 14%NI groups by using Kruskal-Wallis and Mann-Whitney U tests. Second, we grouped the hamsters based on their mild, moderate, and severe infection, analysed differences among them using the Kolmogorov-Smirnov test and determined statistical significance by Mann-Whitney U test. Significance of the means was set at p<0.05. All calculations were performed using SPSS version 20 (SPSS Inc., Chicago).

## Results

### The 3% protein diet induced weight loss and, ultimately, severe malnutrition

**Figure 1:**
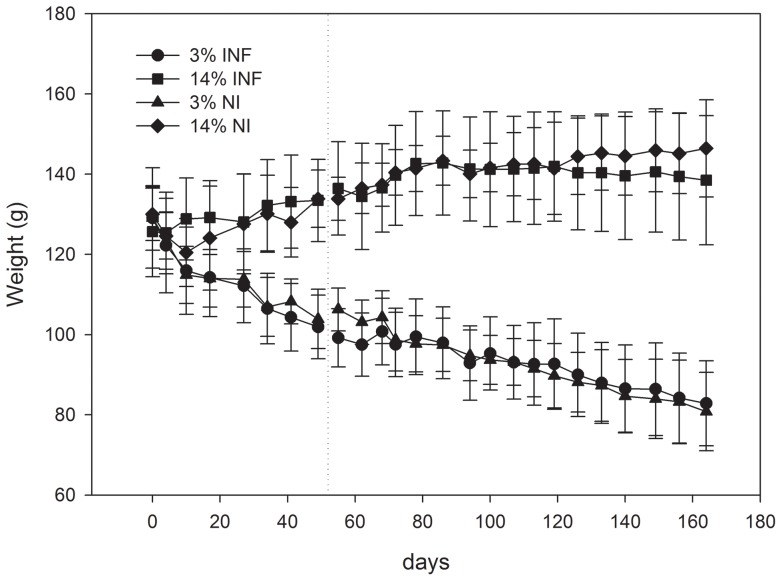
Growth of infected and non-infected hamsters fed the standard (14%) and protein-deficient (3%) diets. Changes in body weight were assessed every seven days. The dotted vertical line indicates the moment of intracardiac infection. Data show means of one representative experiment of two independent assays. The differences in mean body weight between hamsters on the different diets were statistically significant: *p<0.05, **p<0.01, ***p<0.001.

Feeding the animals a diet poor in protein content led to significant alterations in their body weight (Figure 1). These differences were significant (p<0.05) at the moment of infection and throughout the study. Following 166 days of dietary restriction and 113 days after *L. infantum* infection, the weight change percentages were (mean ± standard error) −35.81%±16.38% for the 3%INF group, 10.21%±19.63% for the 14%INF, −37.38%±12.64% for the 3%NI group, and 12.64%±13.77% for the 14%NI group. The hamsters' weight change could, therefore, be considered unrelated to *L. infantum* infection.

The degree of malnutrition of the hamsters, according to Gomez's classification of human malnutrition [18], was determined based on their weight-for-age (WA) results, using the 14%NI group as the control. The WA was calculated according to Anstead et al. [6] as: (*weight of the animals [by group]/expected weight of these animals if they had followed the diet of the 14%NI animals) x 100%.*

The mean WA was 55.59% for 3%NI group, 97.85% for 14%INF, and 56.98% for 3%INF. Animals following the 3% protein diet, irrespectively of the presence of *L. infantum* (groups 3%NI and 3%INF), showed severe malnutrition (WA<60%).

### Protein malnutrition and visceral leishmaniasis induced haematological and biochemical alterations

The 3% protein diet was associated with a significant reduction in the number of leukocytes (p = 0.001) (Table 2). Non-infected malnourished hamsters (3%NI) showed significantly reduced numbers of monocytes, granulocytes and lymphocytes, especially CD4^+^ (p = 0.0112) and B cells (p = 0.007), compared to the 14%NI animals. White blood cells depletion, mainly of monocytes (p = 0.007) and granulocytes (p = 0.031), was also seen in the 14%INF animals compared to non-infected animals on the same diet (14%NI) (Table 2). The low protein diet was also associated with reduced MCV and MCH values (p<0.001), as well as increased RDW values.

**Table 2:** Haematological variables (mean ± SD) evaluated in infected (INF) and non-infected (NI) hamsters following the 14% and 3% protein diets.

Haematological variables	Groups:				Physiological values
	3%INF	14%INF	3%NI	14%NI	
Erythrocytes (×10^6^/mm^3^)	8.42±2.37	8.6±1.72	9.35±1.32	8.85±0.72	3.96–10.60
Haemoglobin (g/dL)	12.69±3.32	14.31±2.62	14.63±1.76	14.49±1.35	10.00–19.20
Haematocrit (%)	43.20±12.02	47.22±9.91	48.20±6.72	49.24±4.65	32.90–59.00
MCV (fL)	71.42±3.26^A***^	74.83±1.19^A***^	71.67±0.65^D***^	75.92±1.68^D***^	64.00–77.60
MCH (pg)	21.22±1.22^A**^	22.71±0.59^A**^	21.70±0.52^D**^	22.53±0.55^D**^	20.00–25.80
PLT (×10^3^/mm^3^)	474.00±277.69	377.00±176.55	477.42±149.60	482.50±105.36	200–590
RDW (%)	15.83±1.39^A***,B*^	12.79±0.48^A***^	14.77±0.58^B*,D***^	12.82±0.23^D***^	
Leukocytes (×10^3^/mm^3^)	4.12±2.06	5.28±2.65	3.94±1.29^D***^	6.53±1.74^D***^	3.00–15.00
Lymphocytes	2.15±1.00^A*^	3.23±1.53^ A*^	2.14±1.00^D**^	3.35±0.92^D**^	50–96%
CD4	0.86±0.42	1.16±0.72	0.80±0.36^D*^	1.21±0.35^D*^	
I-E^k+^ (IgG/IgM^+^)	0.94±0.69	1.28±0.73	0.89±0.38^D**^	1.46±0.45^D**^	
Monocytes	0.19±0.14	0.23±0.15^C**^	0.21±0.09^D***^	0.41±0.12^C**,D***^	0–3%
Granulocytes	1.78±1.06	1.83±1.07^C*^	1.48±0.56^D***^	2.83±0.85^C*,D***^	17–35%

The comparison of the groups showed at least one significant difference among the averages of MCV (p<0.0001), MCH (p = 0.001), RDW (p<0.0001), leukocytes (p = 0.008), lymphocytes (p = 0.013), CD4 (p = 0.05), I-E^k+^ (p = 0.029), monocytes (p = 0.002) and granulocytes (p = 0.005). Statistically significant differences are indicated by (A) for 3%INF vs. 14%INF; (B) for 3%INF vs. 3%NI; (C) for 14%INF vs. 14%NI; and (D) for 3%NI vs. 14%NI. Letters with * indicate p<0.05; letters with ** indicate p<0.01; letters with *** indicate p<0.001. Abbreviations: PLT- platelets, MCV- mean corpuscular volume, MCH- mean corpuscular haemoglobin, RDW- red blood cell distribution width.

*L. infantum* infection was associated with a significant increase in ALkP levels, regardless of the diet followed (Table 3). ALT levels were also increased in both infected groups, but only statistically significant in the 14%INF group (p = 0.023). High levels of AST were found in all groups compared to the 14%NI group, but differences were not significant. The 3%NI animals showed reduced levels of albumin and globulin (p<0.001), but infection in the malnourished animals (3%INF) promoted a significant increase in globulins (p = 0.002). However, the infection induced a reduction of albumin levels in those fed with the standard protein diet (14%INF). A significant increase of creatinine was found between infected animals fed with 3% or 14% protein diet (p = 0.006). Malnourished hamsters (3%NI and 3%INF) also showed significantly reduced total proteins and blood urea nitrogen levels compared to the standard protein diet groups (14%NI and 14%INF); however, the reduction in total proteins was less pronounced in the 3%INF animals than in the 3%NI animals (p<0.007). Both the 3% protein diet and *L. infantum* infection were associated with increased glucose levels (p = 0.028 and p = 0.002, respectively).

**Table 3:** Biochemical profiles (mean ± SEM) of the infected (INF) and non-infected (NI) hamsters fed 14% and 3% protein diets.

Biochemical variables	Groups:				Physiologi-cal values
	3%INF	14%INF	3%NI	14%NI	
ALKP (IU/L)	91.73±36.79^B***^	97.00±41.93^C***^	45.25±19.95^B***^	46.50±15.75^C***^	8–187
ALT (IU/L)	70.73±21.21	79.73±17.18^C*^	56.83±17.67	61.75±14.65^C*^	22–128
AST (IU/L)	95.75±43.53	103.27±48.46	100.75±31.18	75.58±22.92	28–122
Creatinine (mg/dl)	0.57±0.14^A**^	0.39±0.16^A**^	0.46±0.10	0.41±0.16	0.40–1.00
Glucose (mg/dl)	121.45±49.28	121.83±26.12^C***^	108.75±36.14^D*^	80.75±24.82^C***,D*^	37–198
Total proteins (g/dL)	5.95±0.42^A**,B***^	6.58±0.55^A**^	4.92±0.34^B***,D***^	6.65±0.67^D***^	5.20–7.00
BUN (mg/dL)	7.09±3.51^A***^	24.75±9.99^A***^	6.92±6.36^D***^	23.92±9.26^D***^	12–26
Albumin (g/dL)	2.47±0.75	2.69±0.46^C*^	2.10±0.23^D***^	3.03±0.34^C*,D***^	2.60–4.10
Globulin (g/dL)	3.47±0.61^B**^	3.79±0.33	2.83±0.28^B**,D***^	3.61±0.40^D***^	2.70–4.20

There was at least one significant difference among the averages of all biochemical parameters, except AST (p<0.02). Statistical differences are indicated by (A) for 3%INF vs. 14%INF; (B) for 3%INF vs. 3%NI; (C) for 14%INF vs. 14%NI; and (D) for 3%NI vs. 14%NI. Letters with * indicate p<0.05; letters with ** indicate p<0.01; letters with *** indicate p<0.001. Abbreviations: ALkP – alkaline phosphatase, ALT – alanine aminotransferase, AST – aspartate aminotransferase, BUN – blood urea nitrogen.

### The cellular response to leishmanial stimulation was impaired in malnourished animals

**Figure 2:**
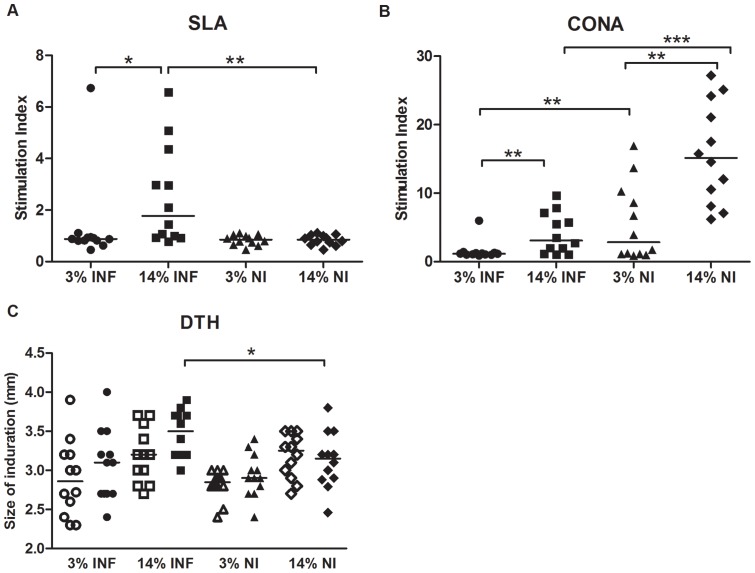
Evaluation of the cellular response and leishmanin test. Lymphoproliferative response to specific SLA (A) and non-specific CONA (B) stimuli, at 113 days post-infection, of PBMCs from infected and non-infected hamsters following the 14% and 3% protein diets. (C) Measurements of the footpads inoculated with PBS (open symbols) and leishmanin (close symbols), taken at 48 h. *p<0.05, **p<0.001. Two independent experiments were performed, one representative experiment is shown.

The response to concanavalin A (CONA) and soluble *Leishmania* antigen (SLA) was assessed using PBMCs from all groups. A statistically significant lymphoproliferative response to SLA was found in well-nourished infected hamsters (P = 0.005). Malnourished animals infected with *L.infantum* (3%INF) showed reduced lymphoproliferative response to both stimuli (Figure 2A and 2B, respectively) (p<0.05). However, infection alone is responsible for decreasing the unspecific lymphoproliferation in hamsters independently of the protein diet consumed (14%INF). Malnutrition also induced a reduced lymphoproliferative response to CONA (3%NI), similar to that showed for the infected well-nourished hamsters (14%INF).

Skin reactivity in response to leishmanin increased significantly in the 14%INF animals (p = 0.022; Figure 2C). Although no significant differences were found, infected 14% hamsters display a more robust DTH response, as compared to infected 3% hamsters.

### Protein malnutrition was associated with a weaker *Leishmania*-specific humoral response

**Figure 3:**
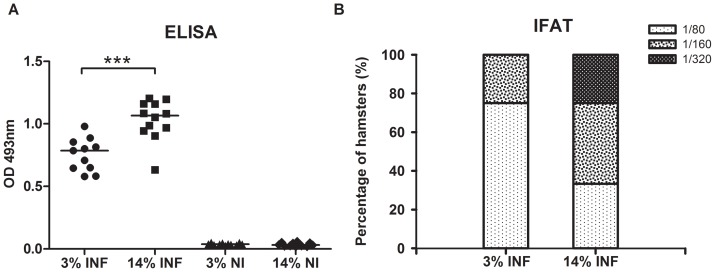
Serum antibodies detected by SLA ELISA (A) and IFAT (B). The difference in response, as measured by ELISA, of the hamsters fed the 3% and 14% protein diets was significantly different. ***p<0.001. Data show medians of one representative experiment of two independent assays.

Anti-SLA antibody levels detected by ELISA were high in both infected groups (Figure 3A). However, the mean IgG level of the 14%INF animals was significantly higher than in the 3%INF group (p = 0.0009). Similar results were obtained in IFAT analysis. Most of the 3%INF animals (8/12) had a 1/80 positive titre, while four had values of 1/160, the maximum titre detected for this group (Figure 3 B). However, higher antibody levels were detected in the 14%INF group; four animals had values of 1/80, five had 1/160 and three had 1/320. IFAT titres of non-infected animals were <1/40.

### *L. infantum* infection induced granulomatous splenitis and hepatitis

**Figure 4:**
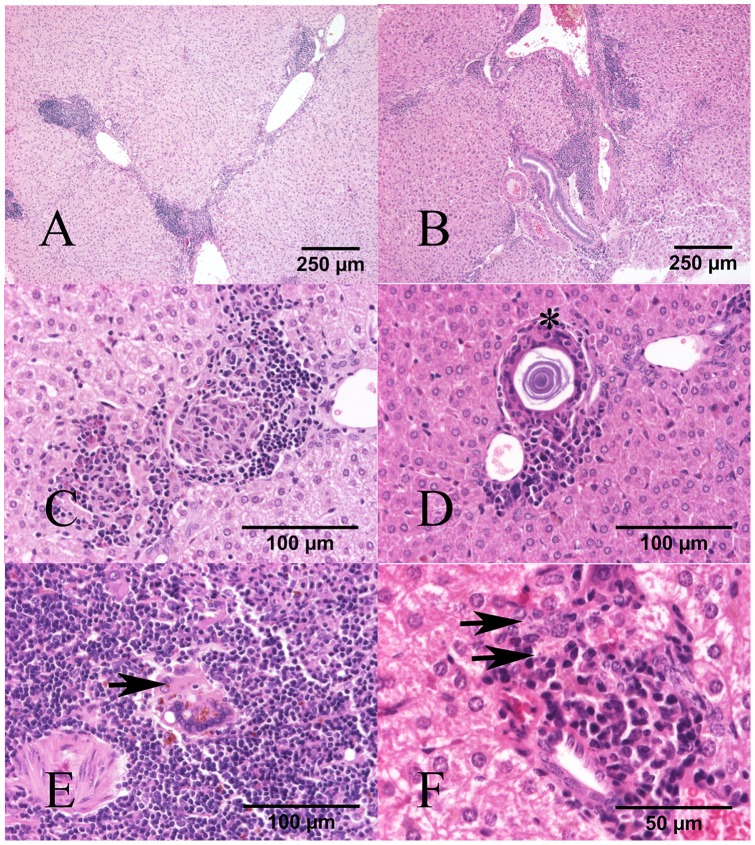
Granulomatous inflammation in liver and spleen of infected hamsters on 3% and 14% protein diets. **H–E.** (A) 3%INF and (B) 14%INF: Severe, multifocal to coalescing granulomatous hepatitis. (C) Liver, 3%INF: Giant multinucleated cell containing a Shaumanns body (asterisk) within a granuloma. (D) Liver, 14%INF: two discrete granulomas containing macrophages, cell debris, epitheliod macrophages, surrounded by few lymphocytes and plasma cells. (E) Spleen, 3%INF: Giant multinucleated cell (arrow) with intracytoplasmic cell debris and hemosiderin. (F) Liver, 14%INF: Granuloma with few macrophages containing rare intracytoplasmic amastigotes (arrows).

Infected hamsters (3% INF and 14% INF) had granulomatous inflammatory infiltrates in liver and spleen. In the liver, these infiltrates were random, multifocal and arranged in either discrete granulomas or sheets of inflammatory cells within the same individual (Figure 4A and 4B). Inflammatory cells consisted of macrophages, lesser lymphocytes, plasma cells, and few giant multinucleated cells (GMCs) that mildly disrupted the parenchyma, and were additionally surrounded by epithelioid macrophages when arranged in granulomas (Figure 4D). Some giant multinucleated cells contained variably sized lamellar concentric mineral concretions often surrounded by a colorless halo (Shaumann's bodies) (Figure 4C). The extent and severity of the granulomatous hepatitis was variable among infected individuals (Table 4). Most of the 3% INF hamsters had mild hepatitis (7/12), while animals in the 14%INF group had moderate inflammation (7/12). In spleens from both infected groups inflammatory infiltrates were either multifocal or diffuse disrupting the totality of the red pulp in more severe cases (Figure 4E). Inflammation was predominantly moderate in both groups (9/12 animals in the 3%INF group versus 7/12 in the 14% INF group). Intralesional *Leishmania* amastigotes were difficult to discern in either organ tissues with routine H-E staining (Figure 4F).

**Table 4:** Relevant histopathological findings.

Observations	Tissue	Groups:			
		3%INF	14%INF	3%NI	14%NI
Multifocal to coalescing granulomatous infiltrates	Liver	+ (7/12)	+ (3/12)	0 (12/12)	0 (12/12)
		++ (3/12)	++ (7/12)		
		+++ (2/12)	+++ (2/12)		
	Spleen	+ (2/12)	+ (2/12)	0 (12/12)	0 (12/12)
		++ (9/12)	++ (7/12)		
		+++ (1/12)	+++ (3/12)		
Giant multinucleated cells with intra-cytoplasmic concentric lamellar mineral	Liver	0 (3/12)	0 (4/12)	0 (12/12)	0 (12/12)
		+ (9/12)	+ (8/12)		

Severity of the histopathological findings: non-existant (0), mild (+), moderate (++), severe (+++).

(n/n) – Number of animals with the given severity of the histopathological feature within each group.

### Malnourishment contributed to higher parasite loads and favoured the development of visceral leishmaniasis

**Figure 5:**
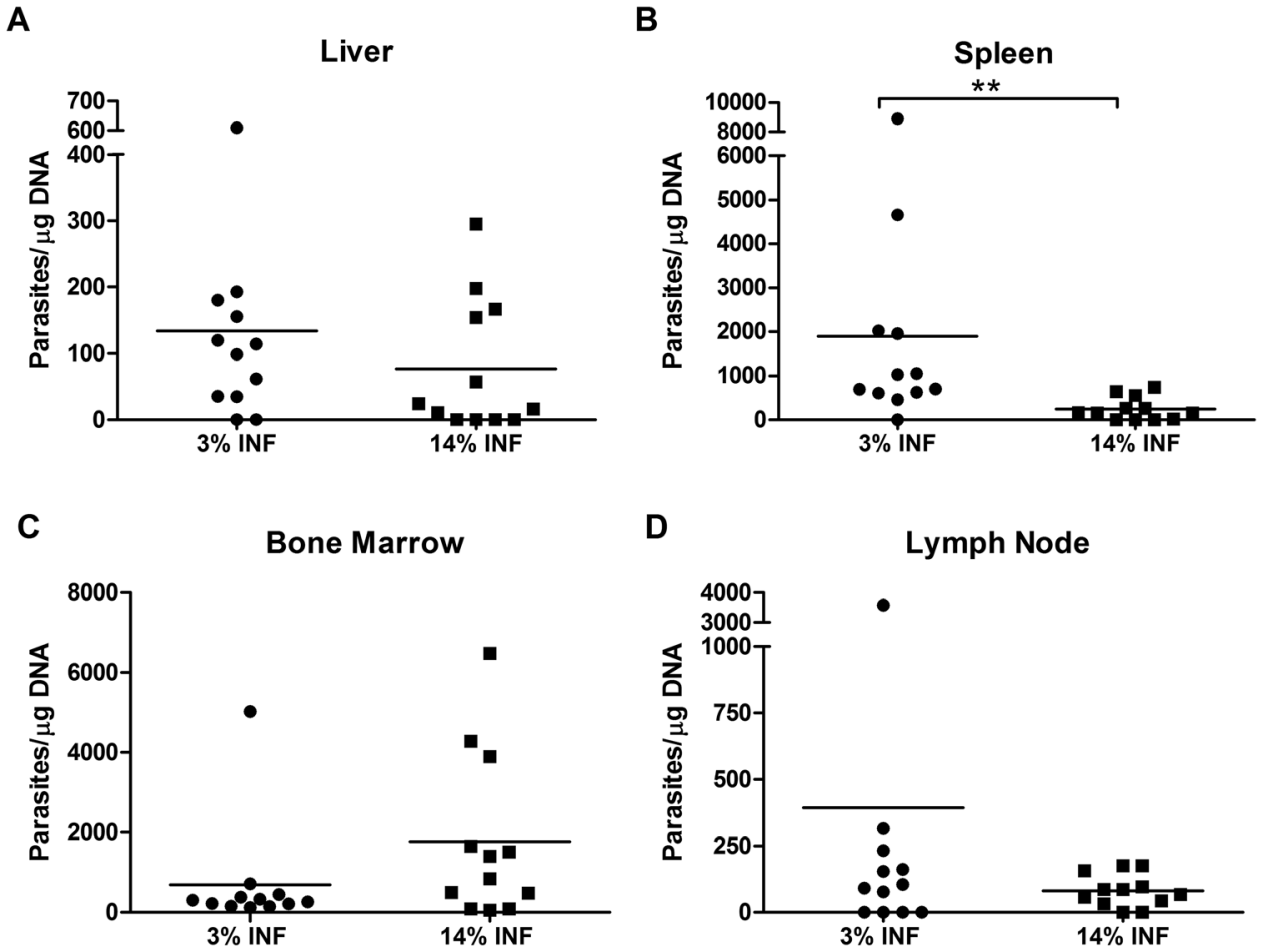
Quantitative distribution of *L. infantum* in malnourished and well-nourished hamsters. The parasite burden was quantified on the (A) liver, (B) spleen, (C) bone marrow, and (D) lymph node. Data represent means ± SD of 12 animals per group of a representative experiment of two independent assays. Splenic parasite loads were significantly increased in malnourished animals. The presence of parasites in blood was detected only in hamsters fed the 3% protein diet (2/12, data not shown).

Parasite loads of blood, skin, bone marrow, lymph node, liver and spleen were measured in all groups (Figure 5). All skin samples were negative for parasites (data not shown). Two out of 12 hamsters in the 3%INF group had parasites in the blood, while no parasites were detected in the blood of any of the 14%INF animals (data not shown). Lymph node's parasite loads were similar in both infected groups, regardless of the diet. In the visceral organs, the 3%INF animals presented higher parasite loads than the 14%INF, with calculated two-fold increase in the liver and seven-fold increase in the spleen (p = 0.0026). However, in the bone marrow, though not statistically significant, the well-nourished hamsters revealed to harbour more parasites than the animals fed with the 3% protein diet.

**Figure 6:**
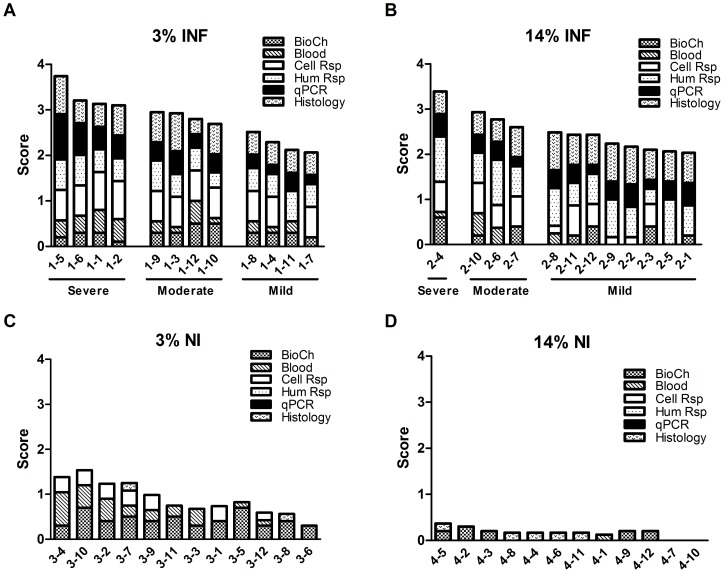
Disease score for visceral leishmaniasis. A score to measure the grade of severity of the disease was performed for 3%INF (A), 14%INF (B), 3%NI (C) and 14%NI (D). For each hamster, a score of 2 was given to values below normal for leukocytes, lymphocytes, PLT and albumin; and to values above normal for total proteins, BUN, AST, globulin and erythrocytes. Other abnormalities in these biochemical (BioCh) or blood variables were scored as 1. Lymphoproliferative responses for SLA and CONA were scored as: 3 for a stimulation index (SI) <0.60; 2 for 0.60≤ SI<2.00; 1 for 2.00≤ SI<4.00; and 0 for SI≥4.00. The humoral response as determined by ELISA was scored as: 1 for 0.10≤ OD 430 nm <0.60; 2 for 0.60≤ OD 430 nm <1.00; 3 for OD 430 nm≥1.00. As determined by IFAT, the humoral response was scored as: 1 for 1/80 titre; 2 for 1/160 titre; 3 for 1/320 titre. Spleen and bone marrow parasite loads as determined by qPCR were scored as: 1 for 35≤ parasites/μg/ml<1000; 2 for 1000≤ parasites/μg/ml<5000; 3 for parasites/μg/ml≥5000. Liver and lymph node parasite loads were scored as: 1 for 35≤ parasites/μg/ml<600; 2 for parasites/μg/ml≥600. Presence of *Leishmania* DNA in the blood was given a disease score of 2. Histopathology for liver and spleen was scored from 0 to 3. A corrective factor was applied to the 6 categories of data to compensate the global score. Visceral leishmaniasis status was recorded as: Mild, when the total score for an animal was 2–2.5; Moderate, when between 2.6–3.0; and Severe, when >3.1. The bars represented each hamster and showed the contribution of each parameter to the total score. Abbreviations: BioCh- serum biochemistry profile, Blood- haematological alterations, Cell Rsp- lymphoproliferative response to SLA and CONA, Hum Rsp- ELISA and IFAT results, qPCR- parasite load (blood, lymph node, spleen, liver and bone marrow), and Histology- grade of alterations in spleen and liver.

Different numbers of animals in the 3%INF and 14%INF groups suffered severe visceral leishmaniasis. By day 165, the 3%INF group had 4 severe, 4 moderate and 4 mild visceral disease; while the 14%INF group had 1 severe, 3 moderate and 8 mild leishmaniasis (Figure 6). The multiple comparison of the total score showed differences among the 3%INF, 14%INF, 3%NI and 14%NI groups (p<0.0001). Particularly, the detailed analysis of the parameters of the score showed that 3%INF and 14%INF presented significant differences in the biochemical parameters, cellular and humoral response (p<0.01). The evaluation of the score classification showed that the proportion of animals with mild visceral leishmaniasis was significantly higher in 14%INF group (p = 0.039). There was at least one significant difference among the averages of the groups mild, moderate and severe on biochemical parameters, cellular response and qPCR (p<0.01). Mildly and moderately infected groups had different biochemical parameters and cellular responses (p<0.02), while mild and severe infections had differences in these two factors and also in the parasite load (p<0.01). Moderately and severely infected hamsters differed statistically in their *Leishmania* burdens (p = 0.007).

## Discussion

In the present study we evaluated for the first time haematological, biochemical, humoral and cellular immune responses, and parasitism in malnourished (3% protein diet) and well-nourished (14% protein diet) hamsters experimentally infected with *L. infantum* after 4 months post-infection.

In the 14%INF group, one severely, three moderately and eight mildly *L.infantum* infected hamsters were recorded. These infected hamsters showed minimal changes in their serum biochemistry in relation to non-infected animals, with the exception of a significant increase of ALT levels. ALKP was also elevated in these well-nourished infected hamsters as it was in the malnourished animals, thus we correlate this parameter to *L. infantum* infection independently of the diet followed. As it is known, most naturally infected persons and dogs are asymptomatic or show subclinical signs of disease [19]–[21]. The opportunity to experimentally mimic some conditions present in the real scenario makes our hamster model a valuable tool for studying the pathological aspects of chronic visceral leishmaniasis, as previously described [22], [23].

It is well known that a state of malnutrition increases the severity of the clinical outcome of leishmaniasis [4], [24]. Using a hamster model of protein malnutrition, we have found that most of the infected animals (3%INF) had a worse manifestation of the disease with four severely and four moderately infected hamsters. This clearly demonstrates the low protein diet ingestion was associated with a higher development of patent visceral leishmaniasis. Although in these infected animals total protein and globulin levels suffered changes in relation to well-nourished hamsters, severe malnourishment produced in non-infected animals most of the alterations of the biochemical parameters, such as reduced total protein, urea, albumin and globulin levels. Low serum albumin and total protein levels are good markers for the protein status of the organism, as previously found in severe malnourished humans and dogs [25]–[26]. Based on these observations, this model provides an excellent opportunity to address the study of the factors implicated in severe malnutrition and, in extension, as showed in this work, in both severe malnutrition and visceral leishmaniasis interaction.

Low white blood cell count is one of the main signs of humans and dogs suffering from visceral leishmaniasis [21], [27]. In our model, *L. infantum* infection caused leukocyte depletion, mainly in monocytes and granulocytes numbers. Furthermore, we found that severe leukopenia was strongly linked to malnourishment, affecting the numbers of all the white cell subsets, including CD4^+^ T lymphocytes and B cells. The malnourished animals – regardless to infection status – also had reduced MCV and MCH values, which are indicative of chronic non-regenerative anaemia. Haematopoiesis is known to be strongly impaired by protein energy malnutrition [28], and the present results suggest that severe protein malnutrition induces bone marrow dysfunction in the hamster, as it does in mice [29]. The presence of *Leishmania* in the bone marrow might also influence haematopoiesis. The inability to develop an efficient, demand-adapted haematopoiesis due to such an infection [30] would influence the final immune response and, therefore, the ability of the host to fight back.

The persistence of *Leishmania* parasites in the lymph nodes was not influenced by protein malnutrition. Increased rates of systemic spread are reported not to depend on higher parasite burdens in the lymph nodes [6]; indeed, the latter authors reported that dissemination was more evident when the total lymph node's parasite burden was lower. However, in our hamsters, protein malnutrition was associated with the presence of circulating parasites and increased parasite burdens, mainly in spleen.

Infected animals had granulomatous hepatitis and splenitis associated with *Leishmania* infection, with only mild insignificant variations in severity between the 3% INF and the 14% INF groups, and regardless of the parasite burdens detected in either tissue. The mere presence of granulomatous inflammation does not necessarily imply an active infection or an effective cellular immune response. As an example, the presence of hepatic granulomas in human visceral leishmaniasis is associated with the maintenance of subclinical infections [31]. Granulomatous inflammation is regulated by multiple factors -such as poorly degradable or persistent antigens and the host's immune response- that determine the effectiveness and type of granulomatous reaction. We could speculate that perhaps some of the immune mechanisms involved against the parasite might have been impaired in the malnourished group, given that a more severe cellular infiltration would have been expected considering the higher parasite loads detected in this group.

Schaumann's bodies are a common finding in granulomatous lesions associated with *Leishmania* [32], to the point that some authors claim that the mere presence of these Schaumann's bodies in the absence of amastigotes is strongly suggestive of *Leishmania* infection in hamsters [33]. These structures were consistently detected in our infected animals in concordance with these observations. The mechanisms involved in the formation of these structures did not appear to be influenced by the protein malnutrition, but by factors related to the infection only.

It is well known that severe protein malnutrition causes functional abnormalities in host defence systems [34], [35], and that the depression of T cell responses following long-term protein malnutrition renders the host unable to prevent the systemic spread of *L. infantum*. Although *Leishmania* infection is known to impair the immune system [5], [36], only two studies have examined the adaptative immune response to *Leishmania* in conjunction with protein restriction. Malafaia et al. [7] reported a reduction in IFN-γ production from SLA-stimulated splenocytes in malnourished mice infected with *L. chagasi* for 4 weeks, while Perez et al. [37] found that protein-deprived mice infected with *L. mexicana* showed a depressed DTH response and a low *in vitro* splenic cell response to SLA and mitogens. In the present work, the 14%INF animals showed a patent specific cellular response, while the 3%INF animals showed an impaired response to leishmanial antigen and lower lymphocyte counts, particularly of CD4^+^ T cells. A DTH response was detected in both the 3%INF and 14%INF animals, but statistically significant differences were only seen between the 14%INF and 14%NI groups. Although most authors report protein energy malnutrition to affect DTH after BCG vaccination [38], some present similar skin test results for malnourished and well-nourished individuals [39].

The ability of the hamsters to mount a cell-mediated immune response when faced with severe malnutrition and visceral leishmaniasis was determined by examining the lymphocyte response to mitogens. In the present hamster model, chronic infection by *L. infantum* induced a decline in T-cell functionality similar in severity to that caused by protein malnutrition, but this condition plus infection dramatically reduced T cell functionality, as reported in a cutaneous leishmaniasis mice model [37]. The humoral immune response was also affected by protein malnutrition, manifested in the form of B cell depletion. Although protein energy malnutrition has been described to affect B cell and antibody responses less profoundly than T cell responses [40], studies on malnutrition in infectious diseases have shown an important depletion of the B cell population [41] as well as antibody production by B cells [42].

## Conclusion

In conclusion, the present work suggests that the nutritional status of individuals infected with *Leishmania* spp. has an important effect on the immune response they can mount, and therefore on the development of visceral leishmaniasis. The present results corroborate epidemiological observations that malnutrition is an important risk factor in the development of severe visceral leishmaniasis. The importance of nutrition as part of immunoprophylactic and therapeutic measures, especially in developing countries, deserves further study.

## References

[1] WHO website. The World Health Report 2002: reducing risks, promoting healthy life, chapter 4. Available: http://www.who.int/whr/2002/en/. Accessed 2014 Jan 27.

[2] Gross RL, Newberne PM (1980) Role of nutrition in immunologic function. Physiol Rev 60: 188–302. 6986619 10.1152/physrev.1980.60.1.188

[3] Mathers CD, Ezzati M, Lopez AD (2007) Measuring the burden of neglected tropical diseases: the global burden of disease framework. PLoS Negl Trop Dis 1: e114. 18060077 10.1371/journal.pntd.0000114 PMC2100367

[4] Cerf BJ, Jones TC, Badaro R, Sampaio D, Teixeira R, et al (1987) Malnutrition as a risk factor for severe visceral leishmaniasis. J Infect Dis 156: 1030–1033. 3680989 10.1093/infdis/156.6.1030

[5] Harrison LH, Naidu TG, Drew JS, de Alencar JE, Pearson RD (1986) Reciprocal relationships between undernutrition and the parasitic disease visceral leishmaniasis. Rev Infect Dis 8: 447–453. 3523702 10.1093/clinids/8.3.447

[6] Anstead GM, Chandrasekar B, Zhao W, Yang J, Perez LE, et al (2001) Malnutrition alters the innate immune response and increases early visceralization following *Leishmania donovani* infection. Infect Immun 69: 4709–4718. 11447142 10.1128/IAI.69.8.4709-4718.2001 PMC98556

[7] Malafaia G, Serafim TD, Silva ME, Pedrosa ML, Rezende SA (2009) Protein-energy malnutrition decreases immune response to *Leishmania chagasi* vaccine in BALB/c mice. Parasite Immunol 31: 41–49. 19121082 10.1111/j.1365-3024.2008.01069.x

[8] Goto H, Lindoso JA (2004) Immunity and immunosuppression in experimental visceral leishmaniasis. 37: 615–623. 10.1590/s0100-879x2004000400020 15064826

[9] Redmond HP, Shou J, Kelly CJ, Schreiber S, Miller E, et al (1991) Immunosuppressive mechanisms in protein-calorie malnutrition. Surgery 110: 311–317. 1650037

[10] Trotter ER, Peters W, Robinson BL (1980) The experimental chemotherapy of leishmaniasis, IV. The development of a rodent model for visceral infection. Ann Trop Med Parasitol 74: 127–138. 6254453 10.1080/00034983.1980.11687322

[11] Melby PC, Chandrasekar B, Zhao W, Coe JE (2001) The hamster as a model of human visceral leishmaniasis: progressive disease and impaired generation of nitric oxide in the face of a prominent Th1-like cytokine response. J Immunol 166: 1912–1920. 11160239 10.4049/jimmunol.166.3.1912

[12] Flavell DJ, Pattanapanyasat K, Lucas SB, Vongsangnak V (1980) *Opisthorchis viverrini*: liver changes in golden hamsters maintained on high and low protein diets. Acta Trop 37: 337–350. 6110324

[13] Yan L, Combs GF Jr, DeMars LC, Johnson LK (2011) Effects of the physical form of the diet on food intake, growth, and body composition changes in mice. J Am Assoc Lab Anim Sci 50: 488–494. 21838977 PMC3148648

[14] Liu H, Steiner BM, Alder JD, Baertschy DK, Schell RF (1990) Immune T cells sorted by flow cytometry confer protection against infection with *Treponema pallidum* subsp. *pertenue* in hamsters. Infect Immun 58: 1685–1690. 2187804 10.1128/iai.58.6.1685-1690.1990 PMC258709

[15] Dondji B, Bungiro RD, Harrison LM, Vermeire JJ, Bifulco C, et al (2008) Role for nitric oxide in hookworm-associated immune suppression. Infect Immun 76: 2560–2567. 18347036 10.1128/IAI.00094-08 PMC2423093

[16] Cunha J, Carrillo E, Sanchez C, Cruz I, Moreno J, et al (2013) Characterization of the biology and infectivity of *Leishmania infantum* viscerotropic and dermotropic strains isolated from HIV+ and HIV− patients in the murine model of visceral leishmaniasis. Parasit Vectors 6: 122. 23622683 10.1186/1756-3305-6-122 PMC3649922

[17] Jungersen G, Jensen L, Rask MR, Lind P (2002) Non-lethal infection parameters in mice separate sheep Type II *Toxoplasma gondii* isolates by virulence. Comp Immunol Microbiol Infect Dis 25: 187–195. 12053916 10.1016/s0147-9571(01)00039-x

[18] Gomez F, Galvan RR, Cravioto J, Frenk S, Santaella JV, et al (1956) Fat absorption in chronic severe malnutrition in children. Lancet 271: 121–2. 13347098 10.1016/s0140-6736(56)90867-4

[19] Chappuis F, Sundar S, Hailu A, Ghalib H, Rijal S, et al (2007) Visceral leishmaniasis: what are the needs for diagnosis, treatment and control? Nat Rev Microbiol 5: 873–882. 17938629 10.1038/nrmicro1748

[20] Sideris V, Papadopoulou G, Dotsika E, Karagouni E (1999) Asymptomatic canine leishmaniasis in Greater Athens area, Greece. Eur J Epidemiol 15: 271–276. 10395058 10.1023/a:1007526401175

[21] Abranches P, Silva-Pereira MC, Conceicao-Silva FM, Santos-Gomes GM, Janz JG (1991) Canine leishmaniasis: pathological and ecological factors influencing transmission of infection. J Parasitol 77: 557–561. 1865262

[22] Requena JM, Soto M, Doria MD, Alonso C (2000) Immune and clinical parameters associated with *Leishmania infantum* infection in the golden hamster model. Vet Immunol Immunopathol 76: 269–281. 11044559 10.1016/s0165-2427(00)00221-x

[23] Garg R, Dube A (2006) Animal models for vaccine studies for visceral leishmaniasis. Indian J Med Res 123: 439–454. 16778322

[24] Gomes CM, Giannella-Neto D, Gama ME, Pereira JC, Campos MB, et al (2007) Correlation between the components of the insulin-like growth factor I system, nutritional status and visceral leishmaniasis. Trans R Soc Trop Med Hyg 101: 660–667. 17442352 10.1016/j.trstmh.2007.02.017

[25] Pointer E, Reisman R, Windham R, Murray L (2013) Starvation and the clinicopathologic abnormalities associated with starved dogs: a review of 152 cases. J Am Anim Hosp Assoc 49: 101–107. 23325600 10.5326/JAAHA-MS-5762

[26] Dusingize JC, Hoover DR, Shi Q, Mutimura E, Kiefer E, et al (2012) Association of serum albumin with markers of nutritional status among HIV-infected and uninfected Rwandan women. PLoS One 7: e35079. 22532840 10.1371/journal.pone.0035079 PMC3331977

[27] Dedet JP, Pratlong F (2003) Leishmaniasis. In: Cook G ZA, editor. Manson's Tropical Diseases. London: Elsevier Science.

[28] Xavier JG, Favero ME, Vinolo MA, Rogero MM, Dagli ML, et al (2007) Protein-energy malnutrition alters histological and ultrastructural characteristics of the bone marrow and decreases haematopoiesis in adult mice. Histol Histopathol 22: 651–660. 17357095 10.14670/HH-22.651

[29] Fock RA, Vinolo MA, Blatt SL, Borelli P (2012) Impairment of the hematological response and interleukin-1beta production in protein-energy malnourished mice after endotoxemia with lipopolysaccharide. Braz J Med Biol Res 45: 1163–1171. 22983177 10.1590/S0100-879X2012007500151 PMC3854220

[30] Takizawa H, Boettcher S, Manz MG (2012) Demand-adapted regulation of early hematopoiesis in infection and inflammation. Blood 119: 2991–3002. 22246037 10.1182/blood-2011-12-380113

[31] Murray HW (2001) Tissue granuloma structure-function in experimental visceral leishmaniasis. Int J Exp Pathol 82: 249–267. 11703536 10.1046/j.1365-2613.2001.00199.x PMC2517779

[32] Laurenti MD, Sotto MN, Corbett CE, da Matta VL, Duarte MI (1990) Experimental visceral leishmaniasis: sequential events of granuloma formation at subcutaneous inoculation site. Int J Exp Pathol 71: 791–797. 2278823 PMC2002383

[33] Gomes-Silva A, Valverde JG, Ribeiro-Romao RP, Placido-Pereira RM, Da-Cruz AM (2013) Golden hamster (*Mesocricetus auratus*) as an experimental model for *Leishmania* (Viannia) *braziliensis* infection. Parasitology 140: 771–779. 23369503 10.1017/S0031182012002156

[34] Faulk WP, Mata LJ, Edsall G (1975) Effects of malnutrition on the immune response in humans: a review. Trop Dis Bull 72: 89–103. 806156

[35] Scrimshaw NS, SanGiovanni JP (1997) Synergism of nutrition, infection, and immunity: an overview. Am J Clin Nutr 66: 464S–477S. 9250134 10.1093/ajcn/66.2.464S

[36] Carvalho EM, Bacellar O, Barral A, Badaro R, Johnson WD Jr (1989) Antigen-specific immunosuppression in visceral leishmaniasis is cell mediated. J Clin Invest 83: 860–864. 2522103 10.1172/JCI113969 PMC303759

[37] Perez H, De La Rosa M, Malave I (1984) The effect of protein restriction on the development of protective immunity to *Leishmania mexicana* . Parasite Immunol 6: 285–294. 6433304 10.1111/j.1365-3024.1984.tb00801.x

[38] Chandra RK (1983) Nutrition, immunity, and infection: present knowledge and future directions. Lancet 1: 688–691. 6132048 10.1016/s0140-6736(83)91980-3

[39] Satyanarayana K, Bhaskaram P, Seshu VC, Reddy V (1980) Influence of nutrition on postvaccinial tuberculin sensitivity. Am J Clin Nutr 33: 2334–2337. 6776793 10.1093/ajcn/33.11.2334

[40] Keusch GT (1981) Host defense mechanisms in protein energy malnutrition. Adv Exp Med Biol 135: 183–209. 6782841 10.1007/978-1-4615-9200-6_10

[41] Taylor AK, Cao W, Vora KP, De La Cruz J, Shieh WJ, et al (2013) Protein energy malnutrition decreases immunity and increases susceptibility to influenza infection in mice. J Infect Dis 207: 501–510. 22949306 10.1093/infdis/jis527 PMC11341849

[42] Law DK, Dudrick SJ, Abdou NI (1973) Immunocompetence of patients with protein-calorie malnutrition. The effects of nutritional repletion. Ann Intern Med 79: 545–550. 4201226 10.7326/0003-4819-79-4-545

